# Knowledge, Attitude, and Practice of Emergency Medical Services Staff in Bleeding Control of Trauma Patients; a Cross sectional Study 

**Published:** 2020-01-26

**Authors:** Yaser Sharifi, Malahat Nikravan Mofard, Maryam Jamsahar, Malihe Nasiri, Mehdi Safari

**Affiliations:** 1 Student Research Committee, School of Nursing and Midwifery, Shahid Beheshti University of Medical Sciences, Tehran, Iran.; 2 Department of Medical Surgical Nursing, School of Nursing and Midwifery, Shahid Beheshti University of Medical Sciences, Tehran, Iran.

**Keywords:** Hemorrhage, advanced trauma life support care, wounds and injuries, health knowledge, attitudes, practice

## Abstract

**Introduction:**

External hemorrhage is a leading cause of preventable death due to trauma and Emergency Medical Services (EMS) staff members play a vital role in the frontline of trauma management. This study aimed to assess the knowledge, attitude and practice of EMS staff in bleeding control.

**Methods:**

This knowledge, attitude and practice (KAP) study was conducted to assess the educational needs of EMS staff of Hormozgan province, Iran, regarding the bleeding control of trauma patients, during 2019. The participants were randomly selected and then their knowledge, attitude, and practice in management of hemorrhage and hemorrhagic shock were assessed using two researcher-made scenario-based questionnaires and one checklist.

**Results:**

The scores for knowledge of the EMS staff regarding actions during complete amputation, status of injured patients, and medical measures during transfer of injured patients were 3.22 ± 0.68, 2.28 ± 0.83, and 2.29 ± 0.62, respectively. The average participants' attitude scores regarding the fear of bleeding, the importance of bleeding control, and confidence in bleeding control were 2.09±0.56, 2.4±0.58, and 1.76±0.55, respectively. The findings indicated that mean practice score was 1.72 ± 0.46 in capillary hemorrhage control, 1.41 ± 0.25 in venous bleeding control, 1.47 ± 0.25 in arterial bleeding control, and 1.56±0.27 in control of bleeding in the amputee limb.

**Conclusion:**

The knowledge, attitude, and practice of EMS staff regarding bleeding control were moderate, positive and appropriate, and incomplete, respectively. Since bleeding is a life threatening status and EMS staff skills are critical in this issue, it seems that we need to provide continuous education in this regard.

## Introduction

Road traffic deaths are expected to increase to 2 million per year by 2020, which will mostly occur in developing countries, especially in South Asian countries (1). Acute, life-threatening hemorrhage after trauma is a major cause of early mortality after trauma, accounting for 40% of trauma deaths globally (2, 3). 

Managing a multiple trauma patient in the presence of severe hemorrhagic shock is naturally a challenge (4). Although active bleeding in the organs or local bleeding may be temporarily controlled by manual pressure or using a tourniquet, an urgent intervention is needed to stop uncontrolled bleeding (5). Traumatic hemorrhagic shock is associated with a high rate of mortality, depending on the duration and extent of reduction in tissue perfusion, most of which occurs in pre-hospital settings (8); and in severe trauma management, the aim of Emergency Medical Services (EMS) is to reduce the time between an injury and definitive medical care. 

 EMS staff act as a bridge between the community and medical services. The EMS is first department in healthcare systems which mainly deal with emergency patients, and the more appropriate, accurate, and faster the services become, the lower the number of deaths and mortality rate and people's confidence to EMS will be increased. 

In Iran, EMS, as an essential element in treatment of emergency patients in the pre-hospital setting, plays a decisive role in reducing mortality and improving patients’ outcome (6). Out of the 31,058 pre-hospital missions accomplished in 2017 in Hormozgan province, Iran, 13,103 were related to road accidents and traumas (7). 

To increase the chance of a patient's survival, several national agencies, including the American Surgeons Committee on Trauma, have attempted to highlight the importance of rapid response to hemorrhage (9). Although the management of bleeding slightly varies between different systems, the overall methods are similar and are in accordance with the general principles of advanced trauma life support (ATLS) protocol (10). 

Identifying the strengths and weaknesses of EMS staff in this regard, and improving the quality of their approach is very important (11). Therefore, this study aimed to evaluate the knowledge, attitude and practice of EMS staff in bleeding control of trauma patients. 

## Methods

***Study Design and Setting***

This knowledge, attitude and practice (KAP) study was conducted during a 1-year period in 2019 to assess the educational needs of EMS staff of Hormozgan province, Iran, regarding bleeding control of trauma patients. The participants were selected from EMS staff of the province and then their knowledge, attitude, and practice in management of hemorrhage and hemorrhagic shock were assessed using two scenario-based questionnaires and one checklist. The study protocol was approved by Ethics Committee of Shahid Beheshti University of Medical Sciences, Iran, receiving the ethical code IR.SBMU.PHARMACY.REC.1398.27. 

***Participants***

All pre-hospital emergency staff members working in the EMS stations of Hormozgan province were selected using census sampling method. Having at least an associate degree in medical emergencies or a bachelor’s degree in nursing, and experience of working in the pre-hospital emergency unit were among the inclusion criteria. The staff members working in the administrative unit of pre-hospital emergency or the dispatch unit were excluded.

***Data Gathering***

The data collection tools developed by the research team included a demographic survey, two scenario-based questionnaires for assessing the knowledge (appendix 1) and attitude, and one checklist for evaluating the practice of EMS staff regarding bleeding control in trauma patients. 

Questionnaires were given to ten experts to assess their face and content validity, which were confirmed after making modifications. To assess the reliability of the questionnaires, they were re-evaluated by 10 experts after a week. The intra-class correlation coefficient (ICC) was 0.9. Also, the reliability of the checklists was assessed using inter-rater agreement coefficient (0.85). 

A demographic survey consisting of 8 items based on personal information and work experience of EMS personnel in the pre-hospital setting was used to gather the data of the participants. The data were collected by an emergency nurse with a master's degree.

In order to collect data, the researcher referred to the pre-hospital emergency units of Hormozgan province. The researcher visited pre-hospital emergency services in Hormozgan, and following coordination with authorities and obtaining consent from the pre-hospital staff members, first, the demographic survey and the knowledge domain questionnaire were given to the participants. Then the attitude domain questionnaire was given to the participants, and the filled out questionnaires were collected. 

Finally, a moulage was used to assess their hemorrhage control skills. After simulating the various vascular injuries on the moulage, emergency personnel were asked to perform remedial measures based on the checklist questions. Then the scores for each section were recorded in the checklist by the researcher. 

***Knowledge assessment tool***

In order to assess the Knowledge level of participants, a questionnaire containing two scenarios in the form of Key Feature Problem (KFP) was used. Scenario 1 consisted of four questions regarding Arterial Bleeding Control and Scenario 2 included three questions regarding the management of amputated injuries. Knowledge of EMS staff in this domain was evaluated and given a score from 0 to 4. The knowledge score of the participants was categorized as low (mean score < 2), moderate (mean score: 2–3), and high (mean score: 3–4).

***Attitude assessment tool***

 A questionnaire consisting of 14 items was used to survey the participants’ attitude toward bleeding and shock control measures. Attitudes questionnaire was set based on a 5-point Likert scale from "totally agree” to “totally disagree". Scores of the participants’ attitude were categorized as follows: negative attitude (mean score: 3.5-5), moderate attitude (mean score: 2.5-3.5), and positive attitude (mean score: 1-2.5).

***Practice assessment*** ***tool***

After simulating various vascular injuries on the moulage, the included EMS staff members were asked to perform remedial measures based on the checklist questions and the scores for each section were recorded in a checklist. The checklist was used to evaluate the hemorrhage control practice including capillary, venous, and arterial hemorrhage, as well as amputation management. Skill of staff in doing each item was calculated as correct receiving 2 points, incomplete or incorrect receiving 1 point, and no practice receiving 0 point. Average point of 1.7 to 2 was considered as good practice level, 1.2 to 1.7 as moderate, and lower than 1.2 as poor.

***Statistical Analysis***

Collected data were analyzed using SPSS software version 21. Mean ± standard deviation or frequency (%) was used for reporting the findings. For investigating the relationship between knowledge, attitude, and practice with demographic characteristics of participants, analysis of variance (ANOVA) was used. P<0.05 was considered as significant.

## Results

***Baseline characteristics of studied EMS personnel***

165 EMS staff members with the mean age of 34.68 ± 5.11 years were evaluated (100% males). The demographic characteristics of the participants are presented in Table 1. 49.1% of the participants were in the 36–45 years age group, 133 (80.6%) were married, and 145 (87.9%) had an associate degree in pre-hospital emergency care. The majority of participants were contractually employed, 90(54.5%) had 11-15 years of working experience in EMS, and 81 (49.1%) had no experience in emergency dispatch.

***Knowledge of participants***

The overall knowledge of participants regarding bleeding control of trauma patients was in the moderate range (2.67 ± 0.39). The knowledge of EMS staff regarding the actions during complete amputation, status of injured patients, and medical measures during the transfer of injured patients were in high (3.22 ± 0.68), moderate (2.28 ± 0.83), and moderate (2.29 ± 0.62) range, respectively (table 2).

There was a significant association between the knowledge levels of EMS staff and age (p = 0.001), marital status (p = 0.039), educational level (p = 0.001), type of employment (p = 0.001), work experience as a dispatcher (p = 0.028), work experience in EMS (p = 0.001), and work experience in hospital (p = 0.004).

***Attitude of participants***

The average attitude score of the participants regarding the fear of bleeding, the importance of bleeding control, and confidence in bleeding control were 2.09±0.56, 2.4±0.58, and 1.76±0.55, respectively (table 3). The ANOVA results demonstrated that there was a significant association between of attitude of EMS staff and type of employment (p = 0.07). 

***Practice of participants***

The total hemorrhage control proficiency score of the participants was 1.54 ± 0.25. The findings indicated that the mean practice score was 1.72 ± 0.46 in capillary hemorrhage control, 1.41 ± 0.25 in venous bleeding control, 1.47 ± 0.25 in arterial bleeding control, and 1.56±0.27 in bleeding control of the amputee limb (table 4).

There was a significant association between the practice levels of EMS staff and age (p = 0.001), marital status (p = 0.001), educational level (p = 0.001), type of employment (p = 0.001), work experience in EMS (p = 0.001), and work experience in hospital (p = 0.001).

**Table 1:** Baseline characteristics of the studied emergency medical services (EMS) staff

**Variable **	**Number (%)**
**Age (year)**	
<25	7 (4.2)
25-35	77 (46.7)
36-45	81 (49.1)
**Educational field**	
Medical emergency	145 (87.9)
Nursing	17 (10.3)
Nurse anesthetist	3 (1.8)
**Education level**	
Associate degree	130 (78.8)
Bachelor	35 (21.2)
**Employment status**	
Contractual	90 (54.5)
Fee for service	47 (28.5)
Fixed term	17 (10.3)
Permanent	11 (6.7)
**Marital status**	
Single	32 (19.4)
Married	133 (80.6)
**Experience of working in disp** **atch ** **(year)**	
No experience	134 (81.2)
<5	29 (17.6)
5-10	2 (1.2)
**Experience of working in EMS (year)**	
<5	58 (35.2)
5-10	22 (13.3)
11-15	81 (49.1)
16-20	4 (2.4)
**Experience of working in hospital (year)**	
No experience	130 (78.8)
<5	35 (21.2)

**Table 2:** Mean Knowledge scores of the studied emergency medical services (EMS) staff members regarding bleeding control Data are presented as mean ± standard deviation

**Items **	**Score**
Examining injured person’s situation	2.28 ± 0.83
Diagnosing the severity of hemodynamic status	2.90 ± 0.92
Immediate measures to protect the injured person	2.58 ± 0.76
Therapeutic measures during the transfer of the injured person	2.29 ± 0.62
Actions in incomplete amputation of the limb	2.90 ± 0.67
Actions in complete amputation of the limb	3.22 ± 0.68
Actions in tightening tourniquet	2.43 ± 0.82
Total	2.67 ± 0.39

**Table 3:** Mean attitude scores of the studied emergency medical services (EMS) staff members regarding bleeding control Data are presented as mean ± standard deviation

**Attitude**	**Score**
Fear	2.09 ± 0.56
Importance of bleeding control	2.4 ± 0.58
Confidence in bleeding control	1.76 ± 0.55
Total	2.08 ± 0.43

**Table 4:** Practice scores of the studied emergency medical services (EMS) staff members regarding bleeding control

**Items**	Categories*			**Mean**
	**2 **	**1**	**0**	
**Capillary bleeding control**				
Investigation of extent of capillary hemorrhage	92.7	7.3	0.0	1.93
Attention to creation of clotting in bleeding site	72.2	27.3	0.0	1.73
No manipulation of bleeding site	33.9	64.2	1.8	1.32
Covering all damaged surface	81.2	17.0	1.8	1.91
**Venous bleeding control **				
Washing or cleaning the area to identify the bleeding site	81.8	18.2	0.0	1.81
Applying direct pressure on site	68.5	31.5	0.0	1.68
Applying pressure on site for 3 min	0	74.5	25.5	0.74
Using pressure patching in case of bleeding continuation	44.2	52.7	3.0	1.41
Not removing the original gauze layer on the site	16.4	75.8	7.9	1.08
Attention to lower limbs for bleeding continuation	72.1	27.9	00.	1.72
**Arterial bleeding control**				
Identifying the exact location of the released artery	71.5	28.5	0.0	1.71
Applying direct pressure on the site	73.9	26.1	0.0	1.74
Squeezing the arterial pressure points in the arm or thigh	3.6	44.8	51.5	0.52
Putting on a tourniquet two fingers above wound	40.6	59.4	0.0	1.4
Not applying the tourniquet on the joint	70.3	29.7	0.0	1.7
Visibility of the wound while tightening the tourniquet	86.7	11.5	1.8	1.84
Loosening the tourniquet after bleeding stops	1.8	40.6	57.6	0.44
Applying pressure on the mild bleeding site and then wound dressing	49.1	49.7	1.2	1.48
Recording the time of putting on a tourniquet	94.5	5.5	0.0	1.94
Visibility of damaged limb	92.7	6.7	0.6	1.92
**Bleeding control in amputated limb**				
Covering the site with sterile gas	44.8	55.2	0.0	1.44
Wrapping crepe bandage twice over the site	10.3	73.3	16.4	0.94
Continuing bandage around the wound	93.3	6.7	0.0	1.93
Visibility of amputated limb	93.3	6.7	0.0	1.93

Data are presented as percentage in each category.

*2: Correct; 1: incomplete or incorrect; and 0: no practice or failed.

**Appendix 1:** Knowledge measuring questionnaire to manage the bleeding and hemorrhagic shock (Please select only four options from the following answers).

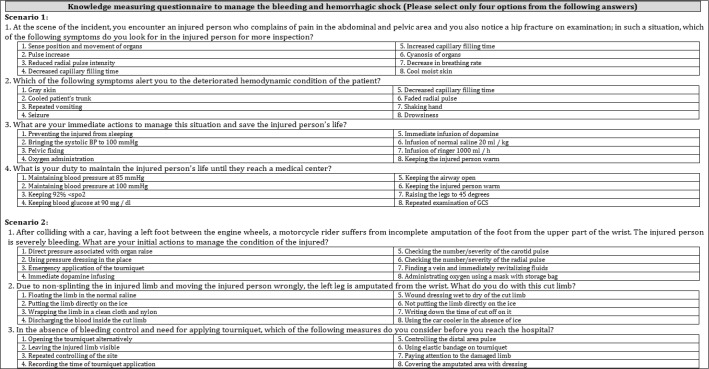

## Discussion

The results of the present study showed that the knowledge, attitude, and practice of the studied EMS staff regarding bleeding control were moderate, positive and appropriate, and incomplete, respectively.

Findings by Ghaffari et al. suggested that the most important educational need of pre-hospital centers is cardiopulmonary resuscitation educational course, and a training course on examining consciousness level of the injured. In addition, three training courses were introduced as high priority courses for hospital emergency care centers, which included courses on how to deal with specific cases (such as amputation and bleeding control), advanced burn training courses, and Advanced Trauma Life Support, all of which had the same degree of importance, followed by self-care educational course in road accidents. The study indicated the necessity for formulating and holding educational programs on the extracted priorities to promote the skills of staff in pre-hospital and hospital centers (12).

In the first scenario of this study, findings indicate that participants had moderate performance in examining injured person’s condition, diagnosis of severity of hemodynamic status, taking immediate measures to protect the injured person, and therapeutic measures during the transfer of the injured person. In addition, regarding the second scenario, their decisions for preserving the incomplete amputated limb and tightening the tourniquet was categorized as average; however, they had better decision-making regarding acting for complete amputation and were ranked as good.

The studied EMS personnel had a positive attitude toward fear of bleeding and shock as well as hemorrhage control, but given the importance of hemorrhage control and its irreparable consequences, more attention should be paid to these skills and EMS personnel need more training in this regard. In their study, Parvaresh et al. compared the effects of scenario-based and lecture-based education methods on knowledge and attitude of emergency medical technicians towards patients with chest trauma and the results showed that in the scenario-based education method, the mean knowledge and attitude scores were significantly higher (p <0.001). Given the impact of scenario-based education and its role in making use of the learner's intellectual abilities and their creativity, scenario-based education seems to be an appropriate alternative to traditional teaching methods (13). 

Regarding hemorrhage control practice, findings indicated that in capillary bleeding control, the highest score belonged to examining the extent of capillary bleeding and the majority of staff paid attention to it. In addition, they often covered the entire surface of injured site correctly. However, paying attention to the clotting at the wound site, and not manipulating the site of bleeding was less considered. The average performance of the emergency personnel was satisfactory.

Regarding venous bleeding control, the findings showed that the majority of staff cleaned or rinsed the area correctly to identify the bleeding area and paid close attention to the lower limbs for the purpose of examining the continuation of bleeding. However, they were less skilled in using pressure patching in case of bleeding and not eliminating the original gauze layer on the site. In addition, despite having sufficient skill in applying pressure on the site, the majority of the staff showed negligence to maintaining pressure on the site for 3 minutes, their average performance was not generally satisfactory. 

Regarding arterial bleeding control, the findings showed that the majority of staff paid close attention to the exact location of the released artery and the use of direct pressure on the site. But they did not show mastery on the use of pressure points for the arm and thighs. In terms of using the tourniquet, they didn't pay attention to tightening the tourniquet and observing the joint. The majority knew that they should expose the injured limb and use the symbol of the tourniquet. However, their performance was poor regarding the last stage of using tourniquet, i.e. loosening the tourniquet, for creating mild bleeding current in order to preserve distal limb. Their average performance was not generally satisfactory.

Regarding bleeding control in amputated limp, it was found that less attention was paid to sterility of dressing on the wound site. In addition, they did not show mastery on bandaging the amputated limb. However, they observed exposure of the amputated limb. Their average performance was not generally satisfactory.

In the study by Oyeniyi et al. in two periods of 2005-2006 and 2012-2103 in Huston Trauma Hospital, role of implementing a multi-purpose package of bleeding control was analyzed. Results indicated that mortality did not correlate with gender and race, and the main factors resulting in death were brain damage and bleeding. By implementing this training package, mortality due to bleeding dropped from 36% to 25% (14).

The results of the Down Port study on pre-hospital controlling of bleeding in traumatic patients indicated that the most common cause of traumatic deaths is bleeding, which usually occurs within three hours of injury. This study indicates that identification of severe bleedings should be performed as part of the primary evaluation on the scene, and potential clinical interventions should be performed as soon as possible. Effective treatment of bleeding in the pre-hospital phase depends on quick identification of severe bleeding, direct anatomic control of vascular damage, bleeding restoration, and transfer to hospital (15).

There is a need for formulating in-service educational courses to eliminate any deficiencies in bleeding control and improve the knowledge of pre-hospital emergency personnel for enhancing decision-making. 

## Limitations

Special job condition and personal problems might have affected the psychological condition of the participants at the time of completing the questionnaire and this might have affected the answers given; however, this was out of the researchers’ control. Additionally, observing the staff when implementing bleeding control skills might have influenced their performance.

## Conclusion:

The knowledge, attitude, and practice of the studied EMS personnel regarding bleeding control were moderate, positive and appropriate, and incomplete, respectively. Since bleeding is a life threatening condition and EMS staff skills are critical in this issue, it seems that we need continuous education in this regard.
